# Safety and efficacy of remimazolam for general anesthesia in elderly patients undergoing laparoscopic cholecystectomy: a randomized controlled trial

**DOI:** 10.3389/fmed.2023.1265860

**Published:** 2023-11-02

**Authors:** Keum Young So, Jihwan Park, Sang Hun Kim

**Affiliations:** ^1^Department of Anesthesiology and Pain Medicine, Chosun University Hospital, Gwangju, Republic of Korea; ^2^Department of Anesthesiology and Pain Medicine, School of Medicine, Chosun University, Gwangju, Republic of Korea

**Keywords:** efficacy, elderly patient, hemodynamics, propofol, remimazolam, safety, sedation

## Abstract

**Introduction:**

There is insufficient evidence regarding the efficacy and safety of remimazolam in elderly patients. Therefore, this study evaluated the differences in the anesthesia characteristics and perioperative hemodynamic profiles of elderly patients receiving total intravenous anesthesia with remimazolam or propofol.

**Methods:**

Eighty-four patients aged >65 years with an American Society of Anesthesiologists physical status of I–III were randomly assigned to Group R (receiving remimazolam, *n* = 42) or Group P (receiving propofol, *n* = 42). In Group R, remimazolam was initiated at a rate of 6 mg/kg/h until loss of consciousness (LOC) was achieved and maintained at 1 mg/kg/h subsequently. In Group P, 1.0–1.5 mg/kg of propofol was injected for 1 min and maintained at 100 μg/kg/min subsequently. The maintenance infusion rate was adjusted to maintain an appropriate depth of anesthesia until the end of the surgery. The primary outcome was the time to LOC. The depth of anesthesia scores and hemodynamic profiles were recorded perioperatively.

**Results:**

The time to LOC was significantly longer in Group R (120 s) than in Group P (60 s) (*p* < 0.001). The time to eye-opening (Group R, 10 min; Group P, 10 min; *p* = 0.056), the incidence of maintenance of hemodynamic changes within 20% of the peri-anesthetic values, and treatments for hemodynamic instability did not differ significantly between the groups. The depth of anesthesia scores did not differ significantly between the groups; however, the scores were higher in Group R than those in Group P before endotracheal intubation. The hemodynamic parameters did not differ significantly at any time point. The time to extubation was longer in Group R (12 min) than that in Group P (10 min) (*p* = 0.007). Similarly, the time to discharge from the operating room was significantly longer in Group R (15 min) compared to Group P (12 min) (*p* = 0.018).

**Conclusion:**

Remimazolam does not exhibit a comparable effect to propofol in terms of anesthesia induction and recovery. However, it demonstrates a similar effect to propofol regarding intraoperative anesthesia depth and hemodynamic profile in elderly patients undergoing remifentanil-based total intravenous anesthesia.

## 1. Introduction

Inhaled anesthetics, intravenous anesthetics or sedatives, and opioids are used by anesthesiologists to induce and maintain general anesthesia. Total intravenous anesthesia (TIVA) is a universal anesthesia management technique performed using intravenous anesthetics or sedatives and opioids. Propofol is a common intravenous sedative that is frequently used in TIVA. However, it can induce serious adverse effects, such as hypotension and bradycardia, delayed recovery, respiratory failure, and propofol infusion syndrome (1, 2). Therefore, there is an increasing need to identify ideal sedatives for TIVA.

Remimazolam, a benzodiazepine that acts on γ-aminobutyric acid (GABA) receptors (1, 3), has specific, unique, and favorable clinical characteristics compared with those of the currently available short-acting anesthetics and sedatives (3, 4). Owing to its structural similarity to remifentanil, it is quickly hydrolyzed by non-specific tissue esterases, resulting in its characteristic rapid onset and offset of sedation, short half-life, and predictable duration of action (1, 4). Similar to other benzodiazepines, the desired levels of sedation with a limited risk of airway obstruction can be achieved more easily with remimazolam, unlike propofol, which has a high ceiling effect (1, 5). In addition, recovery from remimazolam-induced sedation is faster than recovery from sedation induced by other benzodiazepines, and it can be hastened further with the use of flumazenil (4, 5).

Although there are physiological differences between adults and older patients, few studies have evaluated the clinical response to remimazolam in elderly patients (6–8). The time to loss of consciousness (LOC) and extubation after the administration of remimazolam did not show any relationship with age in a previous study (6). However, the time to LOC (TLOC) after the administration of remimazolam was found to be shorter, and the risk of delayed extubation was found to be higher in elderly patients than in adult patients (6, 8). Dose adjustments are not necessary for elderly patients according to manufacturers; however, the rate of administration and dosage should be adjusted carefully based on the patient’s physical condition.

Fast onset and offset of action of sedatives are important; however, minimizing hemodynamic changes is equally important. Several studies have shown that remimazolam is safer than propofol in terms of stable hemodynamic changes (1, 4, 5, 9). Compared with propofol, remimazolam has the advantage of preventing hypotension during the induction of anesthesia and similar anesthetic effects in elderly patients (10, 11). However, there is insufficient evidence regarding the efficacy and safety of remimazolam in elderly patients, and it is unknown whether remimazolam can be used safely and effectively in elderly patients at the clinical doses proposed by manufacturers.

We hypothesized that remimazolam would be as efficient and safe as propofol for the induction, maintenance, and recovery of TIVA. Thus, this study evaluated the anesthesia and hemodynamic profiles during the perioperative period of elderly patients receiving TIVA with remimazolam/remifentanil or propofol/remifentanil. The primary outcome assessed in this study was TLOC after remimazolam or propofol infusion.

## 2. Materials and methods

This prospective, randomized, controlled, single-blind study was approved by the Institutional Review Board of the Chosun University Hospital (Chosun 2021-08-010-001) on September 29, 2021, and prospectively registered with the Clinical Research Information Service (CRIS:^1^ ref.: KCT0006796) on December 2, 2021. This study was conducted in accordance with the Declaration of Helsinki of 1964 and its subsequent revisions.

### 2.1. Inclusions and exclusions

Patients aged >65 years with an American Society of Anesthesiologists physical status (ASA-PS) of I–III who were scheduled to undergo elective laparoscopic cholecystectomy under TIVA between March 24, 2022, and June 30, 2023, were eligible for inclusion in this study. Written informed consent was obtained from all participants or their legal surrogates after providing a thorough explanation of the purpose of the study. Patients with hemodynamic instability, renal or hepatic functional abnormalities, neuromuscular disorders, acute narrow-angle glaucoma, alcohol or drug dependence, or a history of resistance or hypersensitivity to benzodiazepines or other anesthetic drugs were excluded.

### 2.2. Randomizations

Eighty-four patients were randomly assigned to two groups that received either remimazolam (Group R, *n* = 42) or propofol (Group P, *n* = 42). Randomization was performed using a table of random numbers at a 1:1 allocation ratio via a website.^2^ The attending anesthesiologists were responsible for obtaining informed consent from the participants, assigning remimazolam or propofol according to the randomization scheme, and gathering and recording data from the participants. The participants were blinded to the group allocation; however, the attending anesthesiologists were not blinded to the group allocation because of the color difference between the study drugs. All other researchers, except for the attending anesthesiologists, participated in the statistical analysis.

### 2.3. Interventions

In Group R, remimazolam was administered at a rate of 6 mg/kg/h to induce anesthesia until LOC was achieved. After intubation, remimazolam was infused at 1 mg/kg/h and adjusted to a maximum infusion rate of 2 mg/kg/h to maintain an appropriate depth of anesthesia until the end of the surgery. In Group P, 1.0–1.5 mg/kg of propofol was slowly injected for 1 min to induce anesthesia. After intubation, propofol was infused at 100 μg/kg/min and adjusted to maintain an appropriate depth of anesthesia until the end of the surgery. LOC was defined as no response to shaking the patient’s shoulder every 5 s after initiating the administration of the study drug. Appropriate depth of anesthesia was defined as an entropy or bispectral index (BIS) maintained between 40 and 60.

The patients were transferred to the operating room (OR) after premedication with 0.05 mg/kg of intramuscular midazolam. Standard patient monitoring, including electrocardiography, non-invasive blood pressure, end-tidal partial pressure of carbon dioxide, and peripheral pulse oximetry, was initiated prior to the induction of anesthesia. In addition, a device was mounted for train of four (TOF) monitoring using a nerve stimulator to evaluate the degree of muscle relaxation during the surgery.

The attending anesthesiologists anesthetized the patients using TIVA based on the group allocation. After confirming LOC, endotracheal intubation was performed after injecting 0.6–0.9 mg/kg of rocuronium according to the anesthesia management protocol followed at our hospital. Remifentanil was continuously infused at a rate of 0.1–2 μg/kg/min and then maintained within this range after endotracheal intubation. The depth of anesthesia was controlled between 40 and 60 during the maintenance of anesthesia, and the hemodynamic parameters were maintained within 20% of the baseline values (before initiating remimazolam or propofol infusion). Optimal neuromuscular paralysis was maintained under 2 counts of TOF with intermittent injections of rocuronium.

Hypotension, defined as a systolic blood pressure (SBP) of <80 mmHg, was managed with intermittent bolus doses of 100 μg of phenylephrine or 10 mg of ephedrine. Hypertension, defined as an SBP of ≥150 mmHg, was managed with intermittent bolus doses of 1 mg of nicardipine, 60 mg of lidocaine, or 10 mg of esmolol. Bradycardia, defined as a heart rate of <50 beats/min, was managed with intermittent bolus doses of 0.5 mg of atropine. Tachycardia, defined as a heart rate of ≥100 beats/min, was managed with intermittent bolus doses of 10 mg of esmolol or adjustment of the infusion rate of remifentanil, propofol, or remimazolam. An air-forced blanket warmer was used to prevent intraoperative hypothermia.

The infusion of remimazolam, propofol, and remifentanil was discontinued at the end of the surgery after reversing the neuromuscular relaxation with 2 mg/kg of sugammadex. If the return of consciousness was not achieved within 10 min of discontinuing the remimazolam infusion, 0.2 mg of flumazenil was administered, and 0.1 mg of flumazenil was repeatedly administered, if necessary, at the discretion of the attending anesthesiologist. The patients were transferred to the recovery room after the complete reversal of rocuronium-induced neuromuscular paralysis and regaining consciousness.

### 2.4. Outcomes

The primary outcome assessed in this study was TLOC after remimazolam or propofol infusion. The depth of anesthesia score (ADS), SBP, diastolic blood pressure (DBP), mean blood pressure (MBP), and heart rate (HR) were recorded on admission to the operating room (AD_OR), before the infusion of each study drug (before; baseline value), on achieving LOC (LOC), 2 min after LOC (LOC2), after endotracheal intubation (PI), 15 min after anesthesia maintenance (A15), at the end of study drug infusion (ES), and 10 min after ES (ES10).

The time to achieve ADS 50 (TADS50), ADS at ES10, extubation time, and time to discharge from the OR were recorded. The age, sex, height, weight, body mass index (BMI), ASA-PS, comorbid diseases, duration of surgery and anesthesia, cumulative doses of infused remifentanil, use of flumazenil, incidence of maintenance of hemodynamic changes within 20% of the baseline values, and therapeutic interventions for perioperative adverse events were evaluated.

### 2.5. Statistical analysis

G*Power software (ver. 3.1.9.1, Heinrich-Heine-Universität Düsseldorf, Germany) was used to estimate the sample size required to evaluate the primary outcome. The effect size for TLOC was calculated as 0.7 using the mean/standard deviation of the remimazolam group (102/26.6) and the propofol group (78.7/38.4) (12). Sixty-eight patients had to be recruited to achieve 2-tailed statistical significance, α = 0.05, and a power of 80%, with an effect size of 0.7. Considering a dropout rate of approximately 20%, 84 patients were enrolled in this study.

IBM SPSS Statistics for Windows ver. 27.0 (IBM Corp., Armonk, NY, United States) was used for all statistical analyses. Student’s *t*-test was used to analyze continuous variables with normal distribution after the Kolmogorov–Smirnov and Shapiro–Wilk tests. The Mann–Whitney U test was used to analyze continuous variables without normal distribution. The data are presented as mean (95% confidence interval [CI] or median [interquartile range]). The nominal variables were analyzed using the Chi-square or Fisher’s exact test and are presented as numbers (percentage) of patients (*n* [%]). Statistical significance was set at *p* < 0.05.

## 3. Results

**Figure 1:**
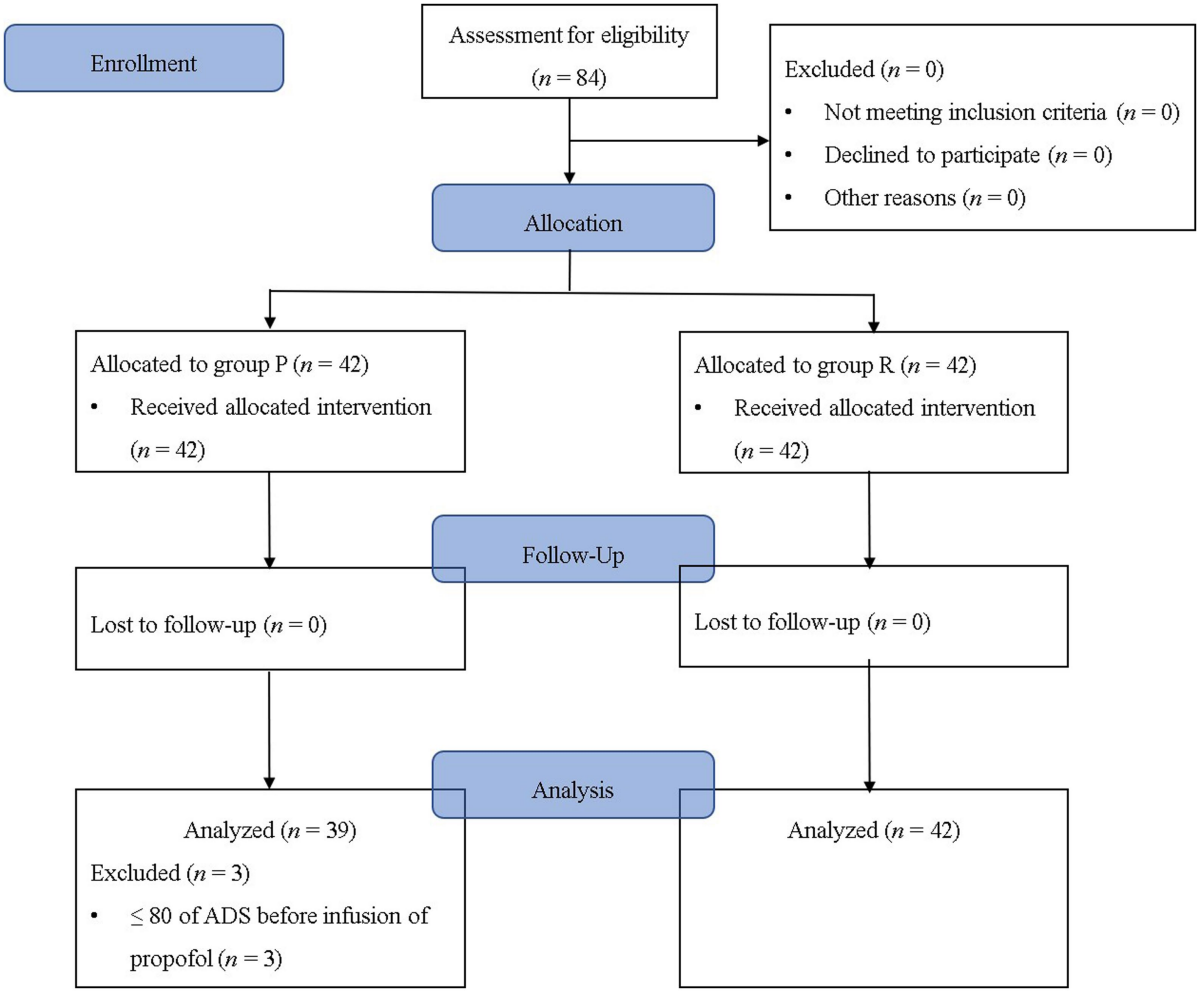
Flowchart of the study. Group P: group receiving propofol, Group R: group receiving remimazolam. ADS, anesthetic depth score.

Among the 84 patients who completed this study without dropping out, three patients from Group P were excluded from the final analysis as they had an ADS of <80 before the infusion of the study drug (Figure 1). The demographic data, except for the history of hypertension, showed no statistically significant difference between the groups (*p* = 0.009; Table 1). Although a statistically significant difference was observed in the history of hypertension, the frequency of pre-anesthetic hypertension, defined as SBP of ≥150 mmHg before anesthesia induction, was not statistically significant (*p* = 0.606; Table 1). The cost difference between propofol and remimazolam exceeded 20-fold (*p* < 0.001; Table 1).

**Table 1:** Demographic data.

	Group P (*n* = 39)	Group R (*n* = 42)	*p* value
Age (year)	76 [70–81]	74.5 [70–78.3]	0.461
Sex (M/F)	22 (52.4)/20 (47.6)	42 (51.9)/39 (48.1)	0.921
ASA-PS (I/II/III)	6 (15.4)/27 (69.2)/6 (15.4)	4 (9.5)/35 (83.3)/3 (7.1)	0.313
Weight (kg)	63 (58.4–67.5)	63.3 (60.1–66.4)	0.910
Height (cm)	160.6 (157.6–163.5)	160.1 (157.6–162.6)	0.803
BMI (kg/m^2^)	24 [20.7–27.5]	24.9 [23.7–26.7]	0.330
Hypertension (yes)	21 (53.8)	34 (81)	0.009*
Preanesthetic hypertension (yes)	25 (59.5)	46 (56.8)	0.606
Diabetes mellites (yes)	14 (35.9)	20 (47.6)	0.285
Respiratory diseases (yes)	2 (4.8)	6 (7.4)	0.303
Surgical duration (min)	35 [25–52]	30 [20–35]	0.153
Anesthesia duration (min)	50 [40–62]	50 [45–55.5]	0.816
Remimazolam (mg)	–	57.5 [50.0–80.0]	–
Propofol (mg)	170.0 [102.0–290.0]	–	–
Cost for study drugs ($)	1.9 [1.2–3.3]	43.7 [38.0–104.0]	< 0.001*
Total dose of remifentanil (μg)	400 [210–700]	500 [250–600]	0.715

Values are expressed as the means (95% confidence intervals), medians [interquartile ranges], or number (percentage) of patients. Group P, group receiving propofol; Group R, group receiving remimazolam; ASA-PS, American Society of Anesthesiologists Physical Status; BMI, body mass index; M, male; F, female. The amount for each drug was calculated in Korean currency, which was converted back to U.S. dollars (exchange rate: 1 Korean won = 0.00076 U.S. dollars, base date: 2023.08.24). *p < 0.05 were considered statistically significant.

TLOC was significantly longer in Group R (120 s) than that in Group P (60 s) (*p* < 0.001; Table 2). TADS50 was significantly longer in Group R (167.5 s) than in Group P (120 s) (*p* < 0.001; Table 2). ADS at ES10 was lower in Group R (66.5) than in Group P (85.5); however, the difference was not significant (*p* = 0.084; Table 2). The time to eye-opening did not differ significantly between Group R (10 min) and Group P (10 min) (*p* = 0.056; Table 2). The time to extubation was longer in Group R (12 min) than in Group P (10 min) (*p* = 0.007; Table 2). The time to discharge from the operating room was significantly longer in Group R (15 min) than in Group P (12 min) (*p* = 0.018; Table 2).

**Table 2:** Time of anesthetic induction and recovery.

	Group P (*n* = 39)	Group R (*n* = 42)	*p* value
TLOC (s)	60 [50–90]	120 [90–134.3]	<0.0001*
TADS50 (s)	120 [77–150]	167.5 [147.5–240]	<0.0001*
ADS at ES10	80 [62–88]	66.5 [51–87.5]	0.084
Eye-opening time (min)	10 [6–12]	10 [9–14.3]	0.056
Extubation time (min)	10 [7–13]	12 [10–15]	0.007*
Time to discharge from OR (min)	12 [10–15]	15 [12–18.5]	0.018*

Values are expressed as the medians [interquartile ranges]. Group P, group receiving propofol; Group R, group receiving remimazolam; ADS, depth of anesthesia score; ES10, 10 min after end of study drug infusion (ES); OR, operating room; TADS50, time to achieve ADS 50; TLOC, time to loss of consciousness (LOC). *p < 0.05 were considered statistically significant.

The incidence of maintenance of the hemodynamic changes within 20% of the peri-anesthetic values did not differ significantly between the groups (Table 3). Treatments for hemodynamic instability based on the study protocol did not differ significantly between the groups (Table 4).

**Table 3:** Incidence of maintenance of hemodynamic changes within 20% of the peri-anesthetic values.

	Group P (*n* = 39)	Group R (*n* = 42)	*p* value
**SBP**			
During AI (yes)	18 (46.2)	14 (33.3)	0.238
After intubation (yes)	22 (56.4)	21 (50)	0.564
During AM (yes)	12 (30.8)	10 (23.8)	0.482
10 Min after anesthesia recovery (yes)	12 (30.8)	10 (23.8)	0.482
**DBP**			
During AI (yes)	25 (64.1)	24 (57.1)	0.522
After intubation (yes)	24 (61.5)	31 (73.8)	0.237
During AM (yes)	16 (41)	23 (54.8)	0.216
10 Min after anesthesia recovery (yes)	31 (79.5)	31 (73.8)	0.547
**MBP**			
During AI (yes)	23 (59)	16 (38.1)	0.060
After intubation (yes)	24 (61.5)	22 (52.4)	0.406
During AM (yes)	11 (28.2)	20 (47.6)	0.072
10 Min after anesthesia recovery (yes)	33 (84.6)	32 (76.2)	0.341
**HR**			
During AI (yes)	33 (84.6)	32 (76.2)	0.252
After intubation (yes)	26 (66.7)	29 (69)	0.819
During AM (yes)	24 (61.5)	27 (64.3)	0.798
10 Min after anesthesia recovery (yes)	22 (56.4)	22 (52.4)	0.716

Values are expressed as the number (percentage) of patients. Group P, group receiving propofol; Group R, group receiving remimazolam; AI, anesthetic induction; AM, anesthesia maintenance; DBP, diastolic blood pressure; HR, heart rate; MBP, mean blood pressure; SBP, systolic blood pressure.

**Table 4:** History of medical treatments during the peri-anesthetic period.

	Group P (*n* = 39)	Group R (*n* = 42)	*p* value
**During induction**			
Local anesthetics (yes)	22 (56.4)	16 (38.1)	0.099
Hypertension Tx. (yes)	1 (2.6)	1 (2.4)	0.734
Hypotension Tx. (yes)	1 (2.6)	2 (4.8)	0.528
Bradycardia Tx. (yes)	1 (2.6)	2 (4.8)	0.528
Tachycardia Tx. (yes)	0 (0)	0 (0)	–
**During maintenance**			
Hypertension Tx. (yes)	2 (5.1)	1 (2.4)	0.472
Hypotension Tx. (yes)	3 (7.7)	3 (7.1)	0.626
Bradycardia Tx. (yes)	1 (2.6)	0 (0)	0.481
Tachycardia Tx. (yes)	0 (0)	0 (0)	–
**During anesthesia recovery**			
Hypertension Tx. (yes)	7 (17.9)	7 (16.7)	0.879
Hypotension Tx. (yes)	2 (5.1)	5 (11.9)	0.248
Bradycardia Tx. (yes)	0 (0)	0 (0)	–
Tachycardia Tx. (yes)	2 (5.1)	6 (14.3)	0.167
Flumazenil Tx. (yes)	0 (0)	3 (7.1)	0.135

Values are expressed as the number (percentage) of patients. Group P, group receiving propofol; Group R, group receiving remimazolam; Tx, treatment.

**Figure 2:**
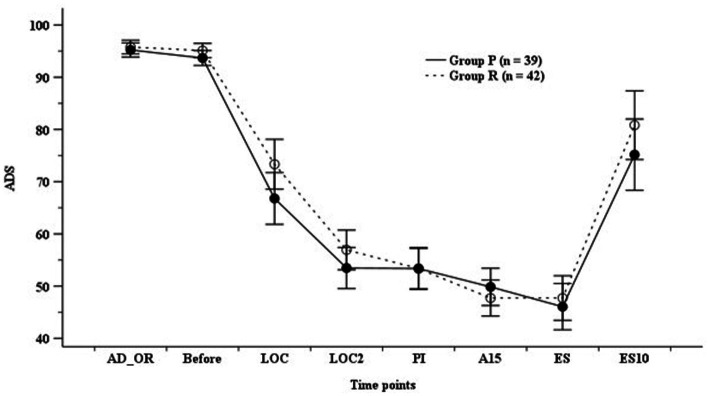
Anesthetic depth score (ADS). Group P: group receiving propofol, Group R: group receiving remimazolam. A15, 15 min after anesthesia induction; AD_OR, administration of operating room; ADS, anesthetic depth score; Before, before infusion of each study drug; LOC, at achieving LOC; LOC2, at 2 min after LOC; ES, at end of study drug infusion; ES10, 10 min after ES; PI, after endotracheal intubation.

**Figure 3:**
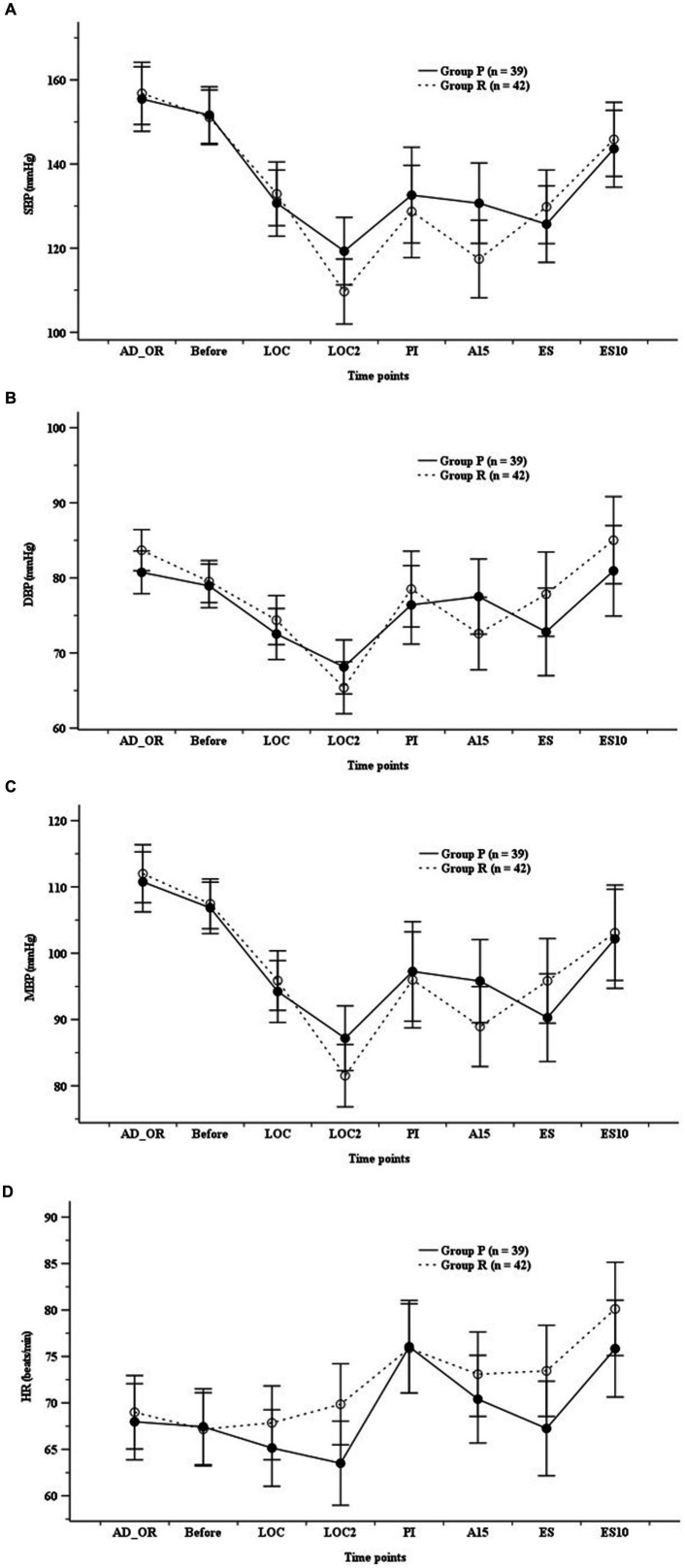
Hemodynamics. **(A)** Systolic blood pressure (SBP), **(B)** diastolic blood pressure (DBP), **(C)** mean blood pressure (MBP), **(D)** heart rate (HR). Group P: group receiving propofol, Group R: group receiving remimazolam. A15, 15 min after anesthesia induction; AD_OR, administration of operating room; ADS, anesthetic depth score; Before, before infusion of each study drug; LOC, at achieving LOC; LOC2, at 2 min after LOC; ES, at end of study drug infusion; ES10, 10 min after ES; PI, after endotracheal intubation.

ADS did not differ significantly between the groups; however, it was higher in Group R than in Group P before endotracheal intubation (Figure 2). The hemodynamic parameters (SBP, DBP, MBP, and HR) did not show any significant differences at any time point (Figure 3).

## 4. Discussion

This study revealed that remimazolam was associated with longer times for LOC, extubation, and discharge from the operating room compared to propofol; however, there was no difference in the duration from the end of anesthesia to eye-opening. In addition, no significant differences were observed between remimazolam and propofol in terms of changes in the depth of anesthesia and hemodynamics.

### 4.1. TLOC and anesthetic depth

Several studies have been conducted comparing the efficacy and hemodynamic stability of remimazolam during anesthesia induction, maintenance, and recovery with those of propofol (12–14). These studies demonstrated that TLOC and recovery were delayed in patients receiving remimazolam compared with those receiving propofol and that remimazolam showed more stable hemodynamic changes. Remimazolam showed a 100% success rate for the induction of anesthesia with an adequate and effective depth of anesthesia at the recommended doses and was non-inferior to propofol as a sedative for general anesthesia (12, 14).

Doi et al. (12) infused remimazolam at a rate of 6 mg/kg/h until achieving LOC, followed by infusion at 1 mg/kg/h to be adjusted as appropriate or administered propofol at 2.0–2.5 mg/kg until achieving LOC followed by infusion at 4–10 mg/kg/h. They reported that TLOC was longer in the remimazolam group (102 ± 26.6 s) than in the propofol group (78.7 ± 38.4 s) and that the mean dose of remimazolam required to achieve LOC during anesthetic induction without the co-administration of remifentanil was 0.17 mg/kg. LOC was confirmed to be an average of 121.2 s in patients who received 6 mg/kg/h of remimazolam without the co-administration of remifentanil during anesthetic induction (15). A recent meta-analysis also reported that TLOC was longer in the remimazolam group than in the propofol group (mean differences = 15.49 s, 95% CI: 6.53–24.46) (13).

Nakanishi et al. (7) reported that LOC and ADS <60 were achieved at 80 s and 200 s, respectively, in elderly patients aged ≥65 years receiving remimazolam infusion (6 mg/kg/h) along with remifentanil infusion (0.25 μg/kg/min) during anesthesia induction. A recent meta-analysis reported that ADS measured using BIS after anesthetic induction was higher in the remimazolam group than in the propofol group (13, 16). Doi et al. (12) also reported that the BIS of the remimazolam group was higher (40.0–82.0) than that of the propofol group (39.0–56.3) during the anesthesia maintenance with remifentanil infusion (12). The current study also showed that TLOC and ADS 50 were higher in the group receiving remimazolam than those in the group receiving propofol during remifentanil-based TIVA; this result is consistent with the results of previous studies.

BIS and the Modified Observer Assessment of Alertness and Sedation (MOAA/S) scores have been used in previous comparative studies on the effects of remimazolam and propofol on the depth of anesthesia (17–20). However, BIS was originally developed for monitoring the depth of propofol-based anesthesia, and there is a weak correlation between benzodiazepine-induced anesthetic depth and BIS (4). Therefore, most authors use the MOAA/S score for evaluating the depth of remimazolam-induced anesthesia. Nevertheless, some studies have suggested that BIS of 60–70 is an appropriate range for remimazolam-induced depth of anesthesia (3, 12, 21–23). Thus, based on the synergy between opioids and benzodiazepines, some authors have suggested that a BIS of <60 could be achieved earlier through the use of opioids with remimazolam and initiation of opioids prior to the initiation of remimazolam (6, 24, 25). Zhao et al. (20) suggested that BIS showed a significant association with the MOAA/S score (*r* = 0.568) and that BIS may be used to predict the state of consciousness in patients receiving remimazolam. The current study also evaluated ADS using BIS or entropy and reported that ADS did not differ significantly despite the higher ADS during anesthesia induction. As mentioned previously, we attributed this finding to the synergistic effects of remimazolam and opioids on ADS.

### 4.2. Anesthetic recovery

The time for eye-opening and extubation was longer in the remimazolam group than that in the propofol group (12, 26). Doi et al. (12) reported that among patients receiving remifentanil infusion during anesthesia maintenance, the time to eye-opening and extubation was longer in the remimazolam group (14.9 min, 19.2 min) than that in the propofol group (10.3 min, 13.1 min). In contrast, Shi et al. (23) reported that among patients receiving remifentanil infusion during anesthesia maintenance and flumazenil (0.5 mg) at the end of the surgery, the time to recovery and extubation was significantly shorter in the remimazolam group than that in the propofol group. A recent meta-analysis reported no differences in the time to eye-opening and extubation between the two groups (13). In the current study, there was no significant difference in the time to eye-opening time and ADS in ES10 between the groups, but there was a significantly longer time to extubation and OR discharge in the group receiving remimazolam compared to the group receiving propofol. Although not shown in the results, except for three patients who received flumazenil in Group R (Table 4), eye-opening time was significantly longer in Group R than in Group P (*p* = 0.037), and ADS was lower in ES10 (*p* = 0.044) when analyzed.

This discrepancy can be explained by the use and difference in the dosages of flumazenil used at the end of the surgery. The use of higher doses of flumazenil hastened recovery from remimazolam (1). Other suggested risk factors associated with delayed recovery are a higher infusion rate and higher BIS after discontinuing remimazolam infusion (6). Although the recovery characteristics of remimazolam showed conflicting results, the recovery time in the group receiving remimazolam was longer than that in the group receiving propofol. However, the difference ranged from 1 to 5 min, which may not be clinically significant in daily practice (1, 12).

### 4.3. Hemodynamics

The greatest advantage of remimazolam is that it has more stable hemodynamic properties than propofol (14, 24, 27, 28). Most intravenous anesthetics exhibit cardiovascular depressive effects by reducing systemic vascular resistance and cardiac contractility in a dose-dependent manner (1). Propofol significantly reduces SBP and DBP in healthy patients, whereas midazolam maintains SBP and DBP. Considering that remimazolam has hemodynamic effects similar to those of midazolam, it may be associated with hemodynamic effects that are more stable than those of propofol.

Studies on continuous maintenance infusion after bolus dosing or continuous infusion with co-administration of remifentanil or sufentanil revealed that compared with propofol, remimazolam is associated with a lower incidence of bradycardia and hypotension (12, 14, 23, 27). Continuous infusion (6 mg/kg/min) of remimazolam resulted in a lower incidence of all and hypotensive adverse drug reactions (39.3, 21.3%) compared with those of propofol (61.3, 50.7%) and remifentanil at the same time (12). The bolus injection (0.3 mg/kg) of remimazolam (24%) for anesthesia induction results in a lower incidence of hypotension during anesthesia induction than bolus injections of propofol (44%) and sufentanil (24). In addition, the incidence of intraoperative hemodynamic fluctuations was lower in patients receiving remimazolam than in those receiving propofol along with opioids (14, 27). Fluctuations in MBP, HR, cardiac index, and cardiac output during anesthesia induction were lower in the remimazolam group than in the propofol group, along with opioids, without significant differences (16, 29, 30). A recent meta-analysis reported that remimazolam was associated with a significantly lower risk of post-induction hypotension than propofol in patients undergoing non-cardiac surgery (risk ratio [RR] = 0.59, 95% CI: 0.44–0.78) (13). Wu et al. (28) also reported that the remimazolam group showed better hemodynamic stability with a lower incidence of hypotension (RR = 0.43, 95% CI: 0.34–0.55) compared with that in the propofol group.

However, the current study did not show a significant difference in the hemodynamic or cardiovascular events between the remimazolam and propofol groups. Thus, further research is needed to determine the effects and mechanisms of hemodynamic changes when remimazolam and opioids are used in conjunction in elderly patients.

### 4.4. Limitations

The present study had some limitations. First, this study was conducted using the dose recommended for adults by pharmaceutical manufacturers (6 mg/kg/h), without using the recommended dose of remimazolam for elderly patients, based on previous studies. Although dose adjustment is not necessary for the elderly, TLOC was shorter in elderly patients than in younger patients receiving the same dose of remimazolam, and the dose of remimazolam required to achieve LOC in 95% of patients (ED95) decreased as the age increased (6, 31–33). The ED95 of remimazolam to achieve LOC without the co-administration of opioids within 5 min has been reported to decrease with age, which was suggested as 0.19–0.25 and 0.14–0.19 mg/kg in patients aged 60–80 and > 80 years, respectively (31). Another study reported that the ED95 of remimazolam to achieve LOC within 3 min without the co-administration of opioids in elderly patients was 0.25 mg/kg (95% CI, 0.20–0.29) (33). In the current study, remimazolam was continuously injected at a rate of 6 mg/kg/h until LOC was achieved, and the recalculated bolus dose with TLOC (120 s) was 0.2 mg/kg. This corresponds to the lower limit of the suggested dose in previous studies. However, it can be assumed that LOC can be achieved within 3 min because of the co-administration of remifentanil. Second, nonparametric analysis was performed in this study as most of the data were not normally distributed; therefore, it is important to be careful when interpreting and comparing the results of previous studies with the median values of this study. Third, elderly patients have various underlying diseases, which can affect the anesthesia-related efficacy and safety of remimazolam. Patients with a significantly lower BIS before anesthesia induction were excluded from this study, in consideration of this. Fourth, the small sample size may have affected the results of this study. Fifth, the impact of anesthetics on the occurrence of postoperative delirium is of significant interest to many researchers. However, this study did not assess the occurrence of postoperative delirium. Consequently, we are unable to determine whether remimazolam has a notable effect, either positive or negative, on the incidence of postoperative delirium in comparison to other sedatives. Therefore, considering the limitations of this study, further studies evaluating the efficacy and safety of remimazolam are needed.

Additionally, the attending anesthesiologist was unable to blind the research drugs because of the color difference between the study drugs. Although all researchers have tried to minimize bias in data collection from a research ethics perspective, this cannot rule out the possibility of bias in the data collected during anesthesia management. Therefore, it is necessary to be careful in interpreting the results for remimazolam, and a study of administering placebo drugs together for each drug may be necessary to analyze the effectiveness of the two drugs to minimize data collection bias.

Finally, when considering the use of remimazolam, the cost-effective aspect cannot be ignored. In fact, remimazolam costs more than 20 times higher as much as propofol. However, the authors believe that the high cost of remimazolam is inevitable, given that it is a recently developed drug and is not yet commonly covered by health insurance for all patients. Therefore, it may be somewhat unreasonable to evaluate the cost-effectiveness of remimazolam at this stage. With sufficient research on its efficacy, effectiveness, and safety, and with potential inclusion in health insurance coverage for anesthesia induction and management, there should be further discussions regarding cost-effectiveness evaluation.

## 5. Conclusion

In conclusion, remimazolam does not have a comparable effect to propofol on anesthesia induction and recovery in elderly patients receiving remifentanil-based TIVA. However, the times to LOC, extubation, and discharge from the operating room were longer in the remimazolam group by 1, 2, and 3 min, respectively, although these differences may not be clinically significant. Furthermore, it has a comparable effect to propofol on intraoperative anesthesia depth and hemodynamic profile.

## Data availability statement

The raw data supporting the conclusions of this article will be made available by the authors, without undue reservation.

## Ethics statement

The studies involving humans were approved by the Institutional Review Board of the Chosun University Hospital. The studies were conducted in accordance with the local legislation and institutional requirements. The participants provided their written informed consent to participate in this study.

## Author contributions

JP: Conceptualization, Investigation, Methodology, Project administration, Visualization, Writing – original draft. KS: Conceptualization, Formal analysis, Investigation, Methodology, Project administration, Visualization, Writing – original draft, Writing – review & editing. SK: Conceptualization, Investigation, Methodology, Project administration, Visualization, Writing – original draft, Data curation, Formal analysis, Funding acquisition, Resources, Software, Supervision, Validation, Writing – review & editing.

## Footnotes

^1^https://cris.nih.go.kr/

^2^https://www.graphpad.com/quickcalcs/
